# A Female With Urinary Bladder Leiomyoma: A Case Report

**DOI:** 10.7759/cureus.51326

**Published:** 2023-12-30

**Authors:** Salahadin Lamy, Mohammed F Hadidi, Nawaf Alhamami, Marshad A Almutairi, Abdullah Qashgry

**Affiliations:** 1 Urology, King Abdulaziz Medical City, National Guard Health Affairs, Jeddah, SAU; 2 Urology, King Saud Bin Abdulaziz University for Health Sciences, College of Medicine, Jeddah, SAU; 3 Urology, King Abdulaziz University, College of Medicine, Jeddah, SAU

**Keywords:** bladder mass, turbt, cystectomy, leiomyoma, bladder

## Abstract

Bladder leiomyoma is a rare benign tumor that can be found in different bladder parts. Leiomyomas can be investigated through a computed tomography (CT) scan or magnetic resonance imaging (MRI), along with cystoscopy, and surgical intervention is considered the standard treatment for this type of cancer. Our case is a 36-year-old female who presented to a urology clinic with lower abdominal pain and lower urinary tract symptoms (LUTS) for three months. The patient was investigated using MRI and was found to have intramural bladder leiomyoma, which was treated with partial cystectomy with bladder mass resection as it is the gold standard treatment.

## Introduction

The majority of bladder tumors have their origins in the urothelium and are typically malignant in nature. Conversely, benign mesenchymal tumors arising within the bladder are relatively rare, accounting for only 1-5% of all bladder neoplasms [1-2]. Among these, bladder leiomyoma stands as a rare benign tumor, with an approximate incidence rate of 0.43% among all bladder tumor types and a documented occurrence of approximately 250 cases in the existing literature. Despite their rarity, bladder leiomyomas represent the most prevalent subtype of benign mesenchymal tumors within the bladder, constituting 35% of these. They typically manifest in females during the latter half of their fourth decade, with a notable female predominance, displaying a 3:1 ratio [3-5]. The precise pathophysiological mechanisms underpinning their formation remain a subject of uncertainty. Various theories have been posited to explain their development, including hormonal irregularities, vestigial remnants in the bladder, perivascular inflammation leading to metaplasia of bladder structures, chromosomal aberrations, and bladder wall infection or inflammation [6]. They are typically found in different bladder parts, approximately 30% occurring extravesical, 7% intramurally, and 63% endovesically [7]. The initial diagnostic evaluation often involves procedures such as cystoscopy, ultrasound, computed tomography (CT), or magnetic resonance imaging (MRI); however, the definitive diagnosis is ultimately established through histopathological examination. Surgical intervention constitutes the standard treatment for bladder leiomyomas, and the specific surgical approach is contingent on factors such as tumor size and location within the bladder wall. Small and readily accessible tumors can be managed via transurethral resection of the bladder tumor (TURBT), while tumors situated in less favorable locations may necessitate segmental resection or laparoscopic partial cystectomy [8-10].

## Case presentation

**Figure 1:**
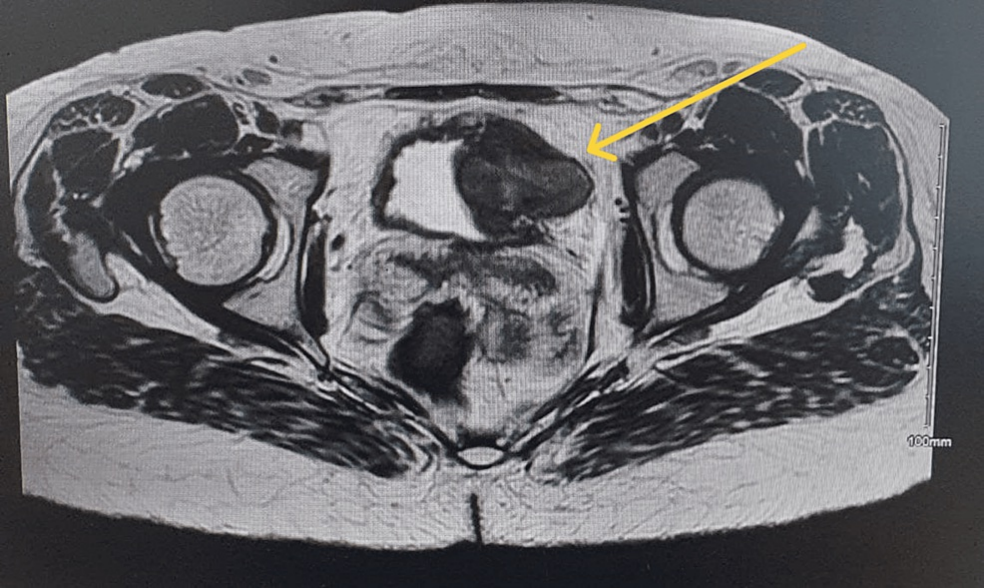
Magnetic resonance urogram in axial view showing intramural left lateral wall bladder lesion 3 cm × 2 cm.

**Figure 2:**
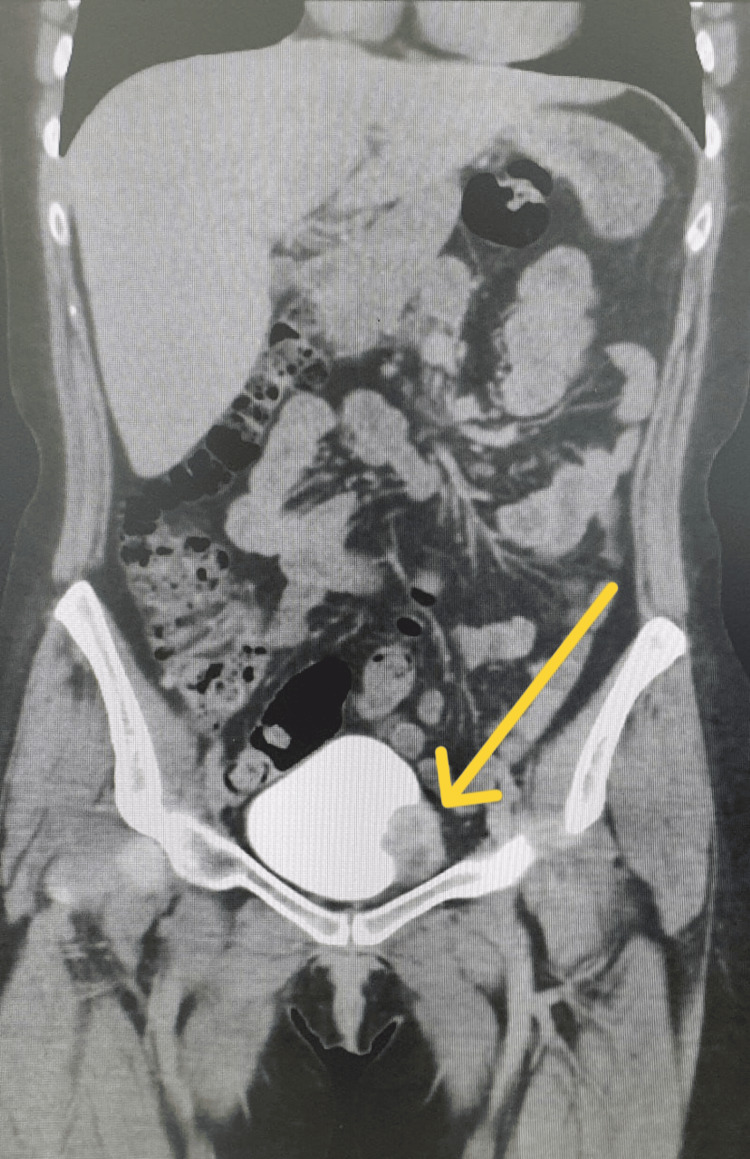
Computed tomography in coronal view showing left lateral wall bladder lesion 3 cm × 2 cm.

This is a case of a 36-year-old female, medically and surgically free, who presented to the urology clinic in another hospital complaining of lower abdominal pain that lasted for three months and was progressive in nature. Associated with lower urinary tract symptoms (LUTS) in the form of a burning sensation and dysuria with no hematuria. However, the patient had no previous surgical or medical history, with an unremarkable systematic review. The patient was treated as a case of urinary tract infection (UTI) in another hospital with no symptomatic improvement. Then, an MRI was done, showing cystitis with intramural bladder leiomyoma and minimal free fluid in Douglas's pouch, as shown in Figure 1. A CT scan demonstrated a left lateral wall urinary bladder mass, as demonstrated in Figure 2. Cystoscopy showed a left lateral wall-invaginated mass with no urothelial lesions. Urine cytology was negative for malignancy. The patient underwent an uneventful open partial cystectomy with bladder mass resection. A dissection was made around the mass with a complete excision of the mass. The bladder was checked, and no other lesions were identified. The patient was discharged home the next day in good condition. The Foley catheter was removed one week after the surgery. The specimen was sent for histopathology correlation, and it confirmed the diagnosis of leiomyoma with the presence of “a well-circumscribed, tan-yellow firm nodular lesion with central infarct-type necrosis and cellular periphery dominated by vaguely spindle cells." The patient presented 26 days post-surgery to the clinic complaining of frequency and nocturia, with a negative urine culture and planned for a trial of behavioural therapy for three months.

## Discussion

Despite the rarity of bladder leiomyoma, it is considered the most common benign mesenchymal tumor of the bladder. Moreover, it has been shown to have a mutual incidence between males and females [4]. However, a literature review done by Goluboff et al. showed that out of 37 patients diagnosed with bladder leiomyoma, 26 (70.3%) were females and 11 (29.7%) were males, with a mean age of 44 years old [5]. Although the exact reason behind female preponderance is still unknown, some authors have reported that the female preponderance is due to frequent exposure to pelvic ultrasound, resulting in the incidental diagnosis of bladder leiomyoma [3]. Furthermore, multiple types of tumors have a specific chromosome abnormality, as deletion of chromosome 7 is the most common cytogenic abnormality related to uterine leiomyoma. As a result, further studies might find out the exact cytogenic abnormality associated with bladder leiomyoma [3]. Also, bladder leiomyoma tends to occur in middle-aged patients, in contrast to our 36-year-old patient in this case report [5]. However, Chen et al. reported a case of bladder leiomyoma in a six-year-old boy [11].

Bladder leiomyoma can be classified as endovesical, extravesical, or intramural. In this report, we presented a case with intramural leiomyoma that is considered rare and represents 7% of all reported cases [4]. However, endovesical tumors are the most common type, and the patients may present with hematuria, dysuria, urinary urgency, and urinary retention, especially if the tumors are pedunculated [3]. On the other hand, our patient was presented in the clinic with progressive lower abdominal pain that lasted for three months as well as lower urinary tract symptoms, such as dysuria and a burning sensation.

As for the diagnosis, bladder leiomyoma is still a rare tumor to detect, even with the availability of ultrasound, CT, and MRI. Moreover, cystoscopy is considered the initial tool that might help to differentiate between intramural tumors and endovesical tumors [5]. In this case report, cystoscopy showed a left lateral wall invaginated mass with no urothelial lesions. However, the best initial diagnostic tool that detects smooth-walled solid lesions is ultrasound, but it was not used in this case because the patient came with MRI findings from another hospital. The CT scan assessed the location of the tumor and revealed a left lateral wall bladder mass. Compared to the other diagnostic modalities, such as CT and cystoscopy, ultrasound provides the best initial imaging technique, giving accurate information about the tumor’s location and its relation to adjacent organs [12]. Despite all the imaging modalities, the definitive diagnostic method is histopathology by biopsy, which demonstrates specific features of bladder leiomyoma, such as spindle-shaped cells, as likewise stated in the histopathology findings of this case [10].

Regarding the management of bladder leiomyoma, surgical resection is the gold standard of treatment. However, the method of bladder leiomyoma resection differs depending on the location of the tumor and its size, either laparoscopic, open, or TURBT. According to the review by Goluboff et al., 38% of the patients were managed by TURBT, whereas 62% of the patients underwent open resection, like our patient, who had open surgery with mass resection and partial cystectomy. In this latter study, 18% of the patients who underwent TURBT required a second operation because of incomplete resection of the mass, while none of the reported patients who had undergone open resection needed a second operation. Overall, the recurrence rate is rare in bladder leiomyoma [5,13].

## Conclusions

Bladder leiomyoma is an uncommon condition, constituting less than 0.5% of all bladder tumors. However, a direct and specific approach included a complete history and physical examination, as well as a thorough assessment starting with ultrasound and ending up with histopathology by biopsy. Furthermore, future investigations and studies can identify cytogenetic abnormalities associated with bladder leiomyoma. Surgical removal is essential for confirming the diagnosis and providing a definitive treatment. Fortunately, these tumors generally have a favorable prognosis post-surgery, showing no signs of malignant transformation. Nevertheless, further research is essential to unravel the pathophysiological aspects of these tumors and establish the most suitable approaches for their management and appropriate follow-up.
